# Lack of an Association of *PD-1* and Its Ligand Genes with Behcet's Disease in a Chinese Han Population

**DOI:** 10.1371/journal.pone.0025345

**Published:** 2011-10-19

**Authors:** Qianli Meng, Haike Guo, Shengping Hou, Zhengxuan Jiang, Aize Kijlstra, Peizeng Yang

**Affiliations:** 1 Department of Ophthalmology, Guangdong Eye Institute, Guangdong General Hospital, Guangdong Academy of Medical Sciences, Guangzhou, People's Republic of China; 2 The First Affiliated Hospital of Chongqing Medical University, Chongqing Key Laboratory Of Ophthalmology, Chongqing, People's Republic of China; 3 Eye Research Institute Maastricht, Department of Ophthalmology, University Hospital Maastricht, Maastricht, The Netherlands; Institut Jacques Monod, France

## Abstract

**Background:**

Behcet's disease is a chronic, multi-systemic autoimmune disease. *Programmed cell death 1* (*PD-1*) gene is one of non-human leucocyte antigen genes. It has been demonstrated to be associated with several autoimmune diseases. However, only a few studies have addressed the association of ligand genes of *PD-1*, *PD-L1* and *PD-L2* with autoimmune disease. The purpose of this study was to analyze the potential association of the *PD-1* and its ligand genes with Behcet's disease in a Chinese Han population.

**Methodology/Principal Findings:**

Four single-nucleotide polymorphism (SNPs) rs2227981 and rs10204525 of *PD-1*, rs1970000 of *PD-L1* and rs7854303 of *PD-L2* were genotyped in 405 Behcet's patients and 414 age-, sex-, ethnic-matched healthy controls using polymerase chain reaction-restriction fragment length polymorphism assay. The results revealed that there were no significant differences in the genotype and allele frequencies of *PD-1* rs2227981 and rs10204525 between the Behcet's patients and controls. A similar result was found for *PD-L1* rs1970000 versus healthy controls. Only the C allele and the CC genotype of *PD-L2* rs7854303 were identified in patients and controls. Stratification analysis based on gender and clinical findings did not show any associations between *PD-1* or its ligand polymorphisms and Behcet's disease.

**Conclusions/Significance:**

None of the currently studied SNPs, *PD-1* rs2227981 and rs10204525, *PD-L1* rs1970000 and *PD-L2* rs7854303, are associated with the susceptibility to Behcet's disease in a Chinese Han population. More studies are needed to confirm these findings in Behcet's patients with other ethnic backgrounds.

## Introduction

Behcet's disease is a chronic autoimmune disease characterized by uveitis, recurrent oral aphthae, genital ulcerations and multiform skin lesions [1]. It is quite common in countries along the ancient ‘Silk Road’ that extends from the Far East to the Mediterranean Sea, such as China, Japan and Turkey [1]–[3]. Although the precise pathogenesis of Behcet's disease remains unclear, extensive studies suggest that an autoimmune response and genetic factors are both involved in this disease. The *human leucocyte antigen B51 (HLA-B51)* gene is demonstrated to be the strongest indicator for this disease in a variety of ethnic groups [4]–[7], but it only partly accounts for the genetic predisposition to Behcet's disease. Therefore, the role of non-human leucocyte antigen genes, especially those regulating the immune response, has recently been investigated by various laboratories. Immune response genes such as the *tumor necrosis factor alpha (TNF-α)* gene [8], *SUMO4* gene [9] and *IL-23R* gene [10], have been reported to be associated with Behcet's disease.

*Programmed cell death 1* (*PD-1*) gene is one of non-human leucocyte antigen genes, located in chromosome 2q37.3 and plays an important role in the regulation of the immune response [11]. The ligands for *PD-1* gene were identified as *programmed cell death-1 ligand 1* (*PD-L1*) and *programmed cell death-1 ligand 2* (*PD-L2*), both of which are located in 9p24 [11]. PD-1 and its ligands belong to the CD28:B7 family. PD-1 contains an immunoreceptor tyrosine-based inhibiting motif (ITIM) and an immunoreceptor tyrosine-based switch motif (ITSM) [11], which is expressed on activated T cells, B cells, and myeloid cells [11]. PD-L1 is expressed in T cells, B cells, macrophages, dendritic cells (DCs), and non-lymphoid cells, whereas PD-L2 is observed on activated macrophages, DCs and bone marrow-derived mast cells [11]. Interaction of PD-1 with its ligands could inhibit T-cell receptor-mediated proliferation and cytokine production [11], [12]. *PD-1* deficiency results in the development of a lupus-like disease or a dilated cardiomyopathy in animal models [13], [14]. In mice undergoing anterior chamber–associated immune deviation, we found that both mRNA and protein of PD-1, PD-L1 and PD-L2 were markedly upregulated and CD4^+^PD-1^+^ T cells exhibited antigen-specific suppressive activity [15]. All these results suggest that PD-1 and its ligands are involved in the regulation of the immune response.

Studies on the *PD-1* gene have demonstrated that the polymorphisms of this gene are associated with several autoimmune diseases including systemic lupus erythematosus (SLE) [16]–[20], rheumatoid arthritis (RA) [21], type I diabetes [22], [23], multiple sclerosis [24], ankylosing spondylitis (AS) [25], and Graves' disease (GD) [26], although there are also some conflicting results [27]–[32]. However, only few studies have addressed the association of ligand genes of *PD-1*, *PD-L1* and *PD-L2*, with autoimmune disease [23], [33], [34].

In this study, we extended earlier studies on the association of *PD-1*, *PD-L1* or *PD-L2* gene polymorphisms with autoimmune disease, and investigated whether the single-nucleotide polymorphisms (SNPs) rs2227981 and rs10204525 of *PD-1*, rs1970000 of *PD-L1* and rs7854303 of *PD-L2* could contribute to the development of Behcet's disease in a Chinese Han population.

## Results

The clinical characteristics of the enrolled Behcet's patients were summarized in Table 1. The average age of the Behcet's patients was 32.9±8.9 and that of healthy controls was 31.4±12.5. All Behcet's patients with uveitis had recurrent oral aphthae. The second most common extraocular clinical manifestation was skin lesions, followed by genital ulcerations. Other abnormalities included a positive pathergy test and arthritis. The distribution of genotype frequencies of each SNP in all subjects did not show any significant deviation from the Hardy–Weinberg equilibrium (HWE).

**Table 1:** Clinical features of the investigated patients with Behcet's disease.

	Patients with Behcet's disease	
Clinical features	Total (n = 405)	%
Age at onset (years ± SD)	32.9±8.9	—
Male	337	83.2
Female	68	16.8
Uveitis	405	100
Oral aphthae	405	100
Skin lesions	255	63.0
Genital ulcerations	198	48.9
Positive pathergy test	132	32.6
Arthritis	107	26.4
Hypopyon	88	21.7

Abbreviations: SD, standardized deviation.

Genotype and allele frequencies of the analyzed *PD-1* rs2227981 and rs10204525, and *PD-L1* rs1970000 were depicted in Table 2. Our results showed that there were no significantly differences in the genotype and allele frequencies of rs2227981 and rs10204525 of *PD-1* between the Behcet's patients and controls. A similar result was found for rs1970000 of *PD-L1* versus healthy controls. The frequency of the GG genotype of rs1970000 tended to be lower in Behcet's patients than in healthy controls (0 versus 1.4%, *P* = 0.031). However, no difference was found when the Bonferroni correction was applied (*P*c = 0.279, n = 9).

**Table 2:** Genotype and allele frequencies of *PD-1*, *PD-L1* SNPs in Behcet's patients and healthy controls.

Gene/SNPs	Genotype/allele	Behcet's patients (%) (n = 405)	Healthy controls (%) (n = 414)	*X^2^*	*P*	Odds Ratio (95%CI)
*PD-1*						
rs2227981	CC	234(57.8)	228(55.1)	0.609	0.439	1.116(0.847–1.472)
	CT	151(37.3)	161(38.9)	0.224	0.666	0.934(0.705–1.239)
	TT	20(4.9)	25(6.0)	0.477	0.541	0.808(0.442–1.480)
	C	385(69.2)	389(67.7)	0.332	0.566	1.077(0.838–1.384)
	T	171(30.8)	186(32.3)	0.332	0.566	0.929(0.723–1.194)
rs10204525	AA	214(52.8)	211(51.0)	0.288	0.625	1.078(0.819–1.418)
	AG	163(40.2)	166(40.1)	0.002	1.000	1.006(0.761–1.331)
	GG	28(6.9)	37(8.9)	1.147	0.303	0.757(0.454–1.262)
	A	377(66.4)	377(65.0)	0.240	0.663	1.063(0.833–1.356)
	G	191(33.6)	203(35.0)	0.240	0.663	0.941(0.737–1.201)
*PD-L1*						
rs1970000	TT	326(80.5)	340(82.1)	0.359	0.591	0.898(0.632–1.277)
	GT	79(19.5)	68(16.4)	1.320	0.275	1.233(0.863–1.763)
	GG	0(0)	6(1.4)	5.913	0.031*	—
	T	405(83.7)	408(84.6)	0.170	0.725	0.930(0.658–1.314)
	G	79(16.3)	74(15.4)	0.170	0.725	1.075(0.761–1.519)

* , *Pc* = 0.279 (n = 9).

The *PD-L2* rs7854303 T allele could not be found and only the C allele and the CC genotype were identified in our patients and controls of the Chinese Han population.

As male gender was predominant in Behcet's patients, a stratification analysis according to gender was also performed. The result showed that there was no association of gender with the four SNPs. When the genotype and allele frequencies were analyzed according to the clinical features, our results showed that any of four SNPs was not found to be associated with any of the extraocular findings including oral aphthae, genital ulcerations, skin lesions, positive pathergy test, or arthritis.

## Discussion

This study did not detect an association between Behcet's disease and known polymorphisms of *PD-1* and its ligand genes in a Chinese Han population. The selection of SNPs used in our study was principally based on polymorphisms used in earlier studies. *PD-1* rs2227981 polymorphism occurs in an exon which affects protein synthesis [25]. rs2227981 and rs10204525 of *PD-1* have been found to be associated with SLE, AS or RA [19], [25], [27]. Our previous study has demonstrated that *PD-1* rs2227981 may be negatively associated with the extraocular manifestations of Vogt-Koyanagi-Harada syndrome in a Chinese Han population [32]. Although the *PD-L1* rs1970000 polymorphism locates in intron 4, which does not affect an amino acid substitution, it may be close to or within transcriptional factor-binding sites [23] and may thus influence the expression of the *PD-L1* gene through modifying the binding affinity of transcriptional factors [34]. This polymorphism has been shown to be associated with GD in Japanese patients [34]. *PD-L2* rs7854303 C encodes a serine in the transmembrane region, while *PD-L2* rs7854303 T encodes a phenylalanine. The transmembrane domain may regulate the expression of the protein molecule on the cell surface [35] and plays a role in dimerization or oligomerization of cell surface molecules [36]. The genotype frequency of *PD-L2* rs7854303 T/T has been reported to be significantly increased in patients with SLE [33].

In order to ensure the analysis results, the following attempts were made. First, all genotype distributions of four SNPs in healthy controls were tested and found to be in Hardy–Weinberg equilibrium. Second, the controls and patients were strictly matched according to the gender and the places where they were born to exclude the possible influence of stratification of the population. Behcet's patients of Chinese Han descendents were also strictly selected in this study to avoid the influence of genetic background. Third, a total of 405 Behcet's patients and 414 age-, sex-, ethnically-matched healthy controls were used in this study, and the number of tested samples was large enough to avoid a bias of the results. Finally, 20% of the samples were randomly chosen and analyzed by direct sequencing to validate the genotype findings. In this study, however, we did not find any association of *PD-1* rs2227981 and rs10204525, *PD-L1* rs197000 and *PD-L2* rs7854303 SNPs with Behcet's disease. These results are in disagreement to those seen in other autoimmune diseases reported among different ethnic groups [16]–[27], [33], [34]. This may be explained by the fact that the etiology and pathogenesis of Behcet's disease may be different from other autoimmune diseases [3]. In view of the fact that PD-1 and its ligands are mainly involved in the adaptive immune response, the lack of an association of *PD-1* and its ligands with Behcet's disease presented in this study may provide additional evidence to the current opinion that this disease is an autoinflammatory disease rather than an autoimmune disease [37], [38].

Several previous studies have shown that there is a large variation in the frequencies of *PD-1* polymorphisms among different ethnic groups. In the present study, we found a higher frequency of the *PD-1* rs10204525 A allele in Chinese (65%) than in Caucasican (8.8%–11.5%) [16], [17], [22] and Mexican (46%) [16]. However, the frequency of the *PD-1* rs2227981 C allele in Chinese was comparable with the frequency in Caucasican [16], [17], [22], Mexican [16] and Korean [25] (Table 3). With regards to the association of *PD-L2* rs7854303 polymorphism with autoimmune disease, a study by Wang et al. [33] showed that an rs7854303 polymorphism was positively associated with SLE in Taiwanese patients. Their study revealed that the frequencies of the T/T genotype and T allele were significantly higher in the patients with SLE than that of the controls. The frequencies of the C/C, C/T and T/T genotype were 48.1%, 35.0% and 16.9% respectively, and the C and T allele frequencies were 65.6% and 34.4% in their 160 controls. However, conflicting results were observed among the Chinese Han population. Only the CC genotype and C allele of *PD-L2* rs7854303 were identified in all Behcet's patients and controls in our study, which showed that the polymorphism of *PD-L2* rs7854303 was not associated with Behcet's disease in Chinese Han population.

**Table 3:** Differences in allele frequencies of *PD-1* rs2227981 and rs10204525 among different ethnic groups.

Population controls	*PD-1* rs2227981 C	*PD-1* rs10204525 A
Chinese Han	67.7%	65%
Hong kong Chinese [21]	71%	N/A
Taiwanese [39]	70.5%	68.5%
Korean [25]	60.8%	N/A
Swedish [16]	57%	9%
Spanish [17]	55.3%	11.5%
Danish [22]	60.5%	8.8%
Mexican [16]	61%	46%

N/A, not available.

In summary, to the best of our knowledge, this is the first polymorphism analysis of *PD-1* and its ligand genes with Behcet's disease in a Chinese Han population. This study did not detect an association between Behcet's disease and known polymorphisms of *PD-1* and its ligand genes in a Chinese Han population. A similar result was observed after stratification analysis based on gender and extraocular features. More studies are needed to confirm these findings in Behcet's patients with other ethnic backgrounds and whether other SNPs of *PD-1* and its ligand genes are possibly associated with the susceptibility to Behcet's disease.

## Materials and Methods

### Clinical Samples

Four hundred and five Behcet's disease patients who all belong to the Chinese Han population were recruited in this study. Four hundred and fourteen age-, sex-, ethnically-matched healthy controls were mainly the accompanying persons or spouses of the patients. All patients were recruited from the First Affiliated Hospital of Chongqing Medical University (Chongqing, China) or the Uveitis Study Center of the Sun Yat-sen University (Guangzhou, China). The diagnosis of Behcet's disease was based on the criteria of the International Study Group [40].

### Ethics statement

The protocol was approved by the Ethics Committee of the First Affiliated Hospital of Chongqing Medical University, Chongqing, China (Permit Number: 2009-201004), and written informed consent was obtained from all the study subjects.

### Genomic DNA extraction and genotyping

Genomic DNA was isolated from peripheral blood of patients and controls using the commercial Qiamp DNA Blood Mini Kit (Qiagen, Valencia, CA). Amplification of the target DNA was performed by polymerase chain reaction (PCR). The primers used in this study are presented in Table 4. A 15 µl reaction mixture, which consisted of 7.5 µl GoTaq Green Mater Mix (Promega, Madison, WI), 50 pmoles primers, and 0.2 µg of genomic DNA, was amplified by PCR. PCR conditions were as follows: initial denaturation at 95°C for 3 min followed by 35 cycles of denaturation at 94°C for 30 s, annealing at different temperatures (63°C for rs2227981, rs10204525 and rs1970000, and 58°C for rs7854303) for 30 s, extension at 72°C for 30 s, and a final extension at 72°C for 3 min. The SNPs were genotyped by PCR-restriction fragment length polymorphism (RFLP) analysis. The PCR products of four SNPs were respectively digested with 2 U of Pvu II (Promega, Madison, WI), Hsp92 II (Promega, Madison, WI), Ban II (Promega, Madison, WI) and Mnl I (Fermentas, MBI) restriction enzymes (Table 4) in a 10 µl reaction volume overnight. Digestion products were visualized on 3% agarose gels and stained with GoldView™ (SBS Genetech, Beijing, China). To confirm the accuracy of the method employed, randomly selected subjects (20% of all samples) were analyzed by direct sequencing (Invitrogen Biotechnology Co., Guangzhou, China). Appropriate controls (no template and known genotype) were included in each typing run.

**Table 4:** Primers and restriction enzymes used for PCR-RFLP analysis.

Gene	rs number	SN	Location	Allele	Primers	Restriction enzyme
*PD1*	rs2227981	7785C/T	Exon 5	C	5′-GTGCCTGTGTTCTCTGTGGA-3′ 5′-CCAAGAGCAGTGTCCATCCT-3′	Pvu II
*PD1*	rs10204525	8737A/G	3′-UTR	A	5′-TCAGAAGAGCTCCTGGCTGT-3′ 5′-GGGGAACGCCTGTACCTT-3′	Hsp92 II
*PD-L1*	rs1970000	8923G/T	Intron 4	T	5′-AATGGCTTGTTGTCCAGAGATG-3′ 5′-GTACCACATGGAGTGGCTGC-3′	Ban II
*PD-L2*	rs7854303	47103C/T	Exon 5	C	5′-GCTGCTTCACATTTTCATCCCCT-3′ 5′-CCAGTGCATTGGGTTACCATGA-3′	Mnl I

### Statistical methods

HWE was tested using the χ2 test. Allele frequencies were estimated by direct counting. Allele and genotype frequencies were compared between patients and controls by the χ2 test using SPSS (version 16.0; SPSS Inc., Chicago, IL). The *P* values were corrected (*Pc*) with the Bonferroni correction by multiplying with the number of analyses performed. *P*c<0.05 was considered significant.
